# Three-year outcome of aflibercept treatment for Japanese patients with neovascular age-related macular degeneration

**DOI:** 10.1186/s12886-020-01542-6

**Published:** 2020-07-10

**Authors:** Kanako Itagaki, Tetsuju Sekiryu, Akihito Kasai, Yukinori Sugano, Masashi Ogasawara, Masaaki Saito

**Affiliations:** 1 Department of Ophthalmology, Fukushima Medical University, Fukushima-Shi, Fukushima Pref. Japan; 2 Department of Ophthalmology, Hirosaki University, Hirosaki-Shi, Aomori Pref. Japan

**Keywords:** Aflibercept, Age-related macular degeneration, Polypoidal choroidal vasculopathy, Treat-and-extend

## Abstract

**Background:**

To evaluate the three-year outcome after intravitreal aflibercept injection (IAI) for neovascular age-related macular degeneration (nAMD).

**Methods:**

Forty-nine treatment-naïve nAMD patients (50 eyes) were enrolled in this prospective study. The eyes received IAI at two-month intervals in the first year. The treatment regimen was changed to IAI based on a treat-and-extend approach in the second and third years.

**Results:**

Twenty-nine eyes of 28 patients were successfully followed up over 36 months. The nAMD subtypes included 15 eyes with typical AMD and 14 eyes with polypoidal choroidal vasculopathy. The number of IAIs performed over the 3 years was 17.2 ± 3.1 (mean ± standard deviation). The mean logMAR, which was 0.42 at baseline, improved to 0.19 (*P* = 0.001) at 12 months, and 0.26 (*P* = 0.049) at 36 months. The central retinal thickness (CRT) was 329 ± 120 μm at baseline, 151 ± 38 μm (*P* < 0.001) at 12 months, and 143 ± 61 μm (*P* < 0.001) at 36 months. The mean subfoveal choroidal thickness (SFCT) was 288 ± 97 μm at baseline, 243 ± 82 μm (*P* < 0.001) at 12 months, and 208 ± 63 μm (*P* < 0.01) at 36 months. The changes in logMAR, CRT, and SFCT over the study period did not differ between typical AMD and PCV.

**Conclusion:**

Long-term aflibercept injection can achieve visual improvement and reduce the thickness of the retina and choroid in nAMD. Morphological improvement of these tissues may not be sufficient to sustain earlier visual improvement over the long-term.

## Background

Age-related macular degeneration (AMD), classified as atrophic or neovascular, is a leading cause of blindness in developed countries [1]. Various treatment modalities, such as laser therapy [2], photodynamic therapy [3], and radiotherapy [4], have been developed for AMD. Some of these treatments are applied to treat neovascular AMD (nAMD) in clinics. Anti-vascular endothelial growth factor (VEGF) drug is commonly used in nAMD treatment, and its intravitreal administration has become the first-line treatment. In Japan, pegaptanib, ranibizumab, and aflibercept are anti-VEGF drugs currently approved by the Ministry of Health, Labor, and Welfare. Aflibercept is a fusion glycoprotein that combines the VEGF-binding domains of VEGFR-1 and VEGFR-2, and have approximately 100 times the binding affinity of ranibizumab for VEGF-A, and also have an affinity for VEGF-B and PIGF. Since aflibercept has high VEGF binding activity in the vitreous, it can be used to treat diseased eyes at a lower treatment frequency compared to ranibizumab. Moreover, aflibercept has been reported to provide favorable results for subretinal lesions, such as retinal pigment epithelium detachment [5]. However, these results were based on clinical studies with a follow-up of up to 2 years, and there have been few studies on the long-term results of aflibercept in clinical practice.

Two patterns of anti-VEGF administration schedule have been reported, reactive treatment and proactive treatment. Reactive treatment consists of monthly examinations, and when there is a recurrence finding, the anti-VEGF drug is administered as pro re nata (PRN) treatment [6]. Although the number of anti-VEGF drug injections can be kept to a minimum, the number of hospital visits may increase to regular monthly visits. A disadvantage of reactive treatment is that it is difficult to determine a dosing schedule. It has been reported that long-term visual acuity may decline because the treatment after recurrence could damage the retina [7, 8].

In contrast, proactive treatment is a planned administration, and it is easy for patients to schedule. In addition, the eyes could be treated before a recurrence in proactive treatment. As a proactive treatment, two patterns of administration schedules have been proposed as follows: fixed doses and treat-and-extend (TAE) treatment [9]. In clinical practice, the TAE regimen is often used because the number of visits is reduced compared to a fixed-schedule regimen. Although an advantage of this treatment is that the dosing interval can be adjusted individually according to disease activity, we do not yet fully know the long-term results of this regimen with aflibercept. We herein report the three-year treatment results of aflibercept in patients with treatment-naïve nAMD who received fixed doses in the first year and underwent planned administration, i.e., TAE, in the second and third years.

## Methods

### Patients/patient recruitment

This study, a non-comparative case series, was approved by the Ethics Committee of Fukushima Medical University. It was conducted following the tenets of the Declaration of Helsinki 1975, as revised in 2000. Written informed consent was obtained from all patients to participate in this study. This prospective study enrolled 49 treatment-naïve nAMD patients (50 eyes) after comprehensive ophthalmic examination, such as a decimal visual acuity test, intraocular pressure measurement, slit-lamp, fundus examination, and optical coherence tomography (OCT) examination with mydriasis. Angiography using fluorescein and indocyanine green was performed at baseline to establish the diagnosis and determine the (CNV) types. Written informed consent was obtained from all patients. The registration number of clinical trials is UMIN000010997. Exclusion criteria were a history of retinal angiomatous proliferation, and laser treatment, including photodynamic therapy, vitreous surgery, and/or cataract surgery.

### Treatments

All patients initially received monthly intravitreal aflibercept (2 mg) injection (IAI) three times as a loading dose at the beginning of the treatment, 1 month and 2 months later, followed by a maintenance phase with fixed doses every 2 months for the remaining 8 months, in the first year. In the second and third years, IAI was performed based on a TAE regimen. In the TAE regimen in this study, IAI prolonged the administration interval by 1 month if there were no exudative changes or hemorrhage in the macula. It shortened the injection interval by 1 month if the patient’s eye showed subretinal hemorrhage, retinal edema, or serous retinal detachment. The minimum dosing interval was 1 month, and the maximum dosing interval was 3 months. All patients were treated with IAI monotherapy, and PDT was not performed in this series of patients.

### Ophthalmic examinations

We evaluated visual acuity (logMAR), which was calculated from decimal visual acuity, central retinal thickness (CRT), subfoveal choroidal thickness (SFCT), and the number of IAIs. Spectral-domain OCT (Heidelberg Engineering, Heidelberg, Germany) was used to measure CRT and SFCT at least every 3 months. One of the two medical retina experts (K.I. and M.O.) monitored the patients and decided the IAI plan when they visited the IAI according to the TAE regimen. We did not monitor the patients between the visits for IAI.

### Statistical analysis

A paired t-test was used to analyze the temporal changes in the times of each measurement. The Wilcoxon signed-rank test was applied to compare the results between typical AMD and PCV. *P*-values of less than 0.05 were considered statistically significant.

## Results

Forty-nine patients (50 eyes) were registered for this study, and 28 (29 eyes) (57%) of whom completed the three-year follow-up. Twenty-one patients (43%) were unable to be followed-up for the full 36 months; six patients changed hospital, four patients had cataract surgery, five patients discontinued their visits for unknown reasons, and six patients were excluded from the analysis due to irregular treatment intervals.

Of the 28 patients who were able to receive regular schedule administration and completed the three-year follow-up, 24 were male and four were female, with a mean age of 71.4 ± 8.2 years (mean ± SD). Subtypes of nAMD were typical AMD in 15 eyes (nine eyes with classic CNV and six eyes with occult CNV only) and PCV in 14 eyes (Table 1). The total number of IAI over the study period was 17.2 ± 3.1, 17.4 ± 3.7 in typical AMD, and 17.1 ± 2.6 in PCV (*P* = 0.78). All eyes received aflibercept injections during the first year of follow-up. Furthermore, on average, 4.6 injections (SD, 1.4) in the second year and 4.7 injections (SD, 1.9) in the third year were administered per eye. Twenty-two eyes (75.3%) could extend the IAI interval to 3 months after the final injection.

**Table 1:** Baseline characteristitcs

Eyes/ Patients	29/28
Gender	Male 24 Female 4
Age	71.4 (mean ± standard deviation)
CNV type	Typical AMD 15 eyes (Classic CNV 9 eyes Occult CNV 6 eyes)
	PCV 14 eyes
logMAR baseline	0.416
Central retinal thickness	329±120μm
Subfoveal choroidal thickness	288±97μm

**Fig. 1:**
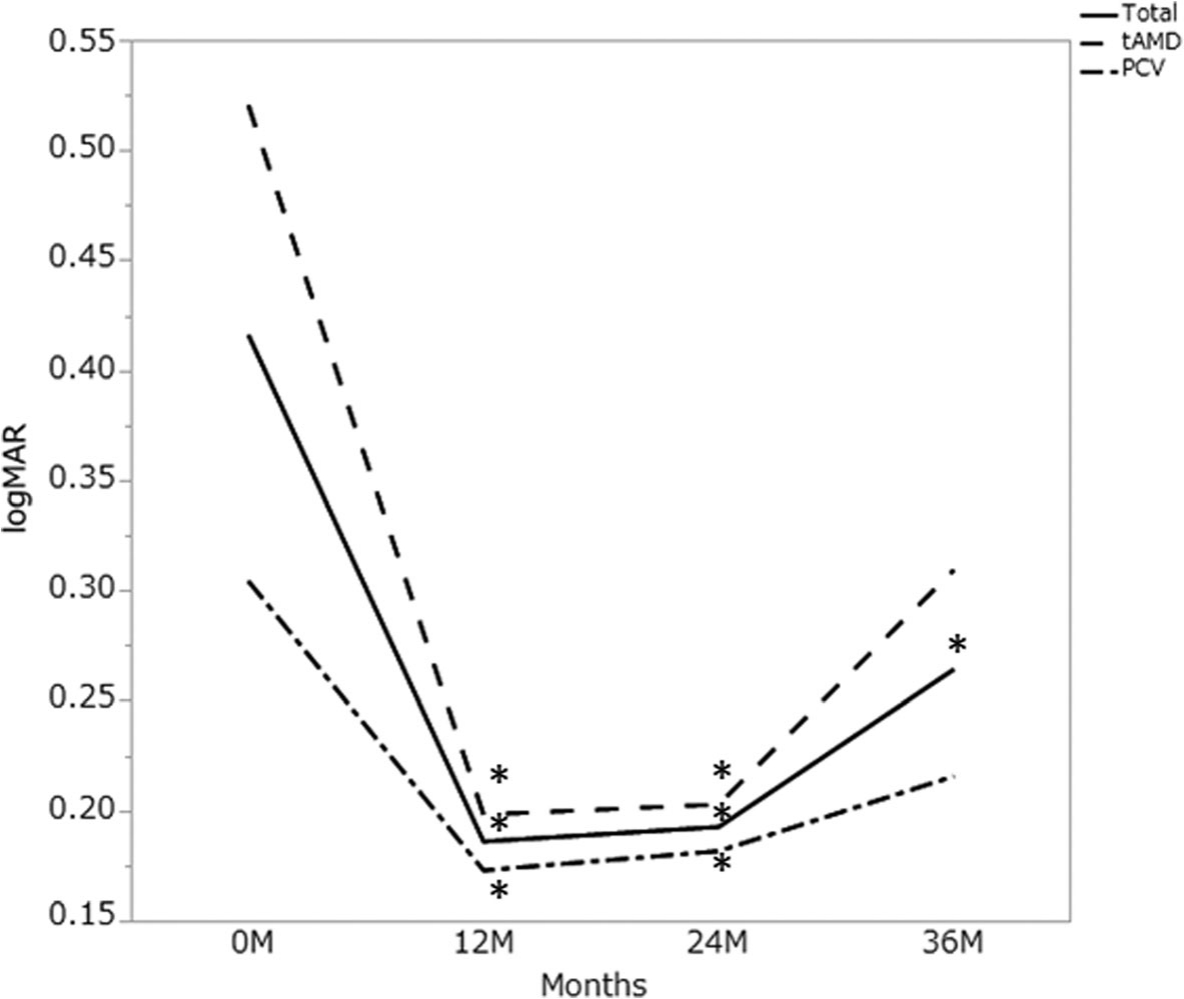
The mean logMAR for 36 months. Total: 29 eyes with neovascular age-related macular degeneration, tAMD: 15 eyes with typical neovascular age-related macular degeneration, PCV: 14 eyes with polypoidal choroidal vasculopathy. Statistical significance between baseline values and each measurement was calculated. *:*P* < 0.05

Overall, visual acuity at 12 months (logMAR 0.19, *P* < 0.001), 24 months (logMAR0.19, *P* < 0.001), and 36 months (logMAR 0.26, *P* = 0.049) were better than those at baseline (logMAR 0.42), although it had significantly decreased at 36 months compared to at 24 months (*P* = 0.049). The mean logMAR in the eyes with typical nAMD was 0.52 at baseline, 0.20 (*P* = 0.007) at 12 months, 0.20 (*P* = 0.007) at 24 months, and 0.31 (*P* = 0.11) at 36 months. The mean logMAR in eyes with PCV was 0.30 at baseline, 0.17 (*P* = 0.022) at 12 months, 0.18 (*P* = 0.043) at 24 months, and 0.21 (*P* = 0.245) at 36 months. LogMAR was also significantly improved over the first two years compared to baseline. The improvement of logMAR from baseline did not reach statistical significance at 36 months in both the typical AMD and PCV groups (Fig. 1).

**Fig. 2:**
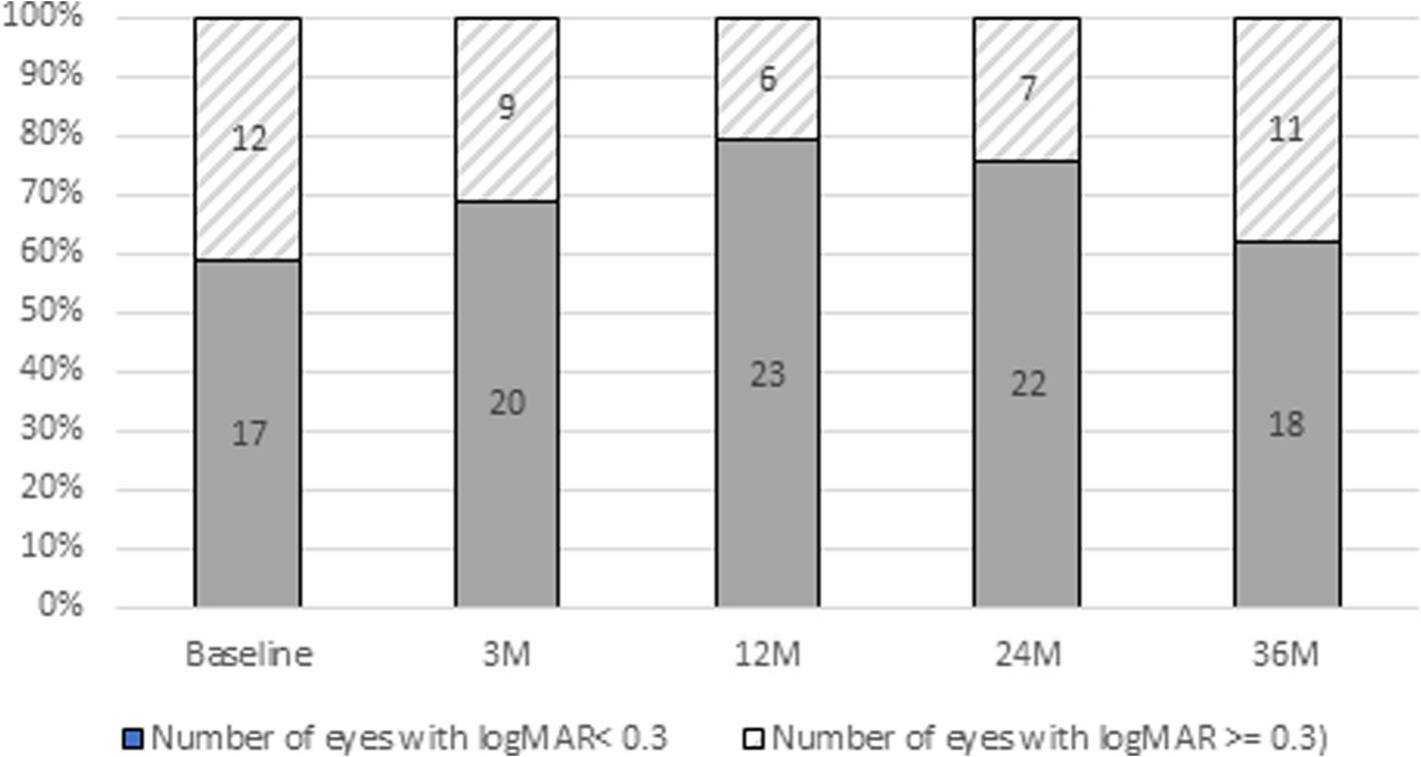
The ratio of eyes with less than logMAR 0.3

For 36 months, 11 eyes (38%) showed improvement of logMAR 0.3 or more. Seven eyes (24%) showed worsening of logMAR 0.3 or less, and 76% of the eyes were able to improve or maintain their vision (Fig. 2).

**Fig. 3:**
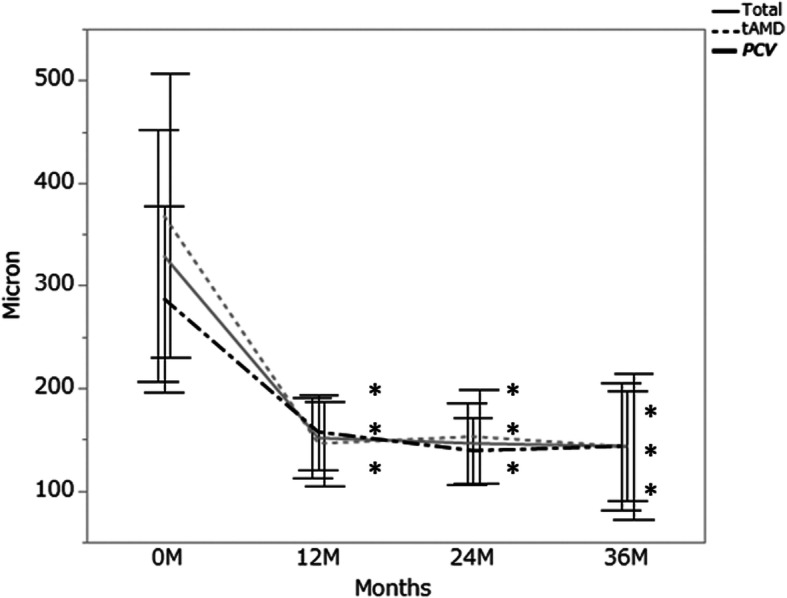
The mean central retinal thickness for 36 months. Error bar, standard deviation. Total: 29 eyes with neovascular age-related macular degeneration, tAMD: 15 eyes with typical neovascular age-related macular degeneration, PCV: 14 eyes with polypoidal choroidal vasculopathy. Statistical significance between baseline values and each measurement was calculated. *:*P* < 0.05

**Fig. 4:**
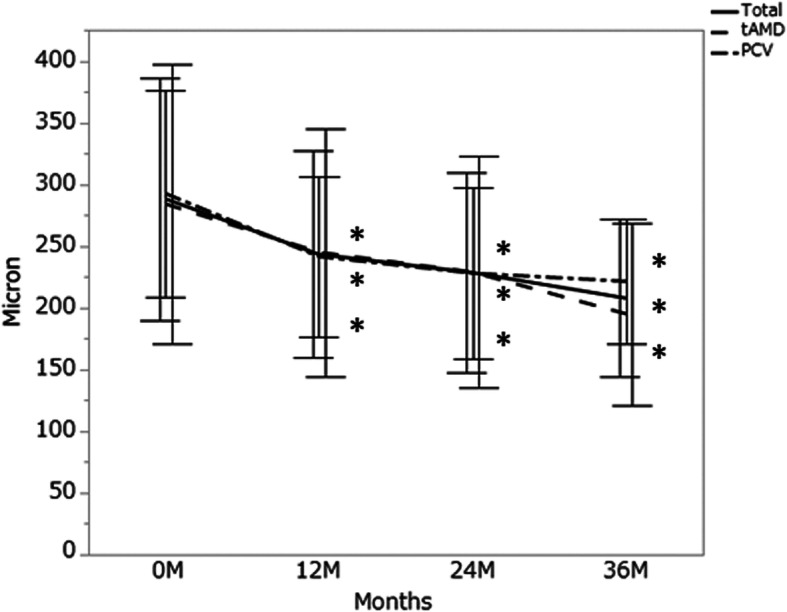
The mean subfoveal choroidal thickness for 36 months. Error bar, standard deviation. Total: 29 eyes with neovascular age-related macular degeneration, tAMD: 15 eyes with typical neovascular age-related macular degeneration, PCV: 14 eyes with polypoidal choroidal vasculopathy. Statistical significance between baseline values and each measurement was calculated. *:*P* < 0.05

The mean CRT was 329 ± 120 μm at baseline, 151 ± 38 (*P* < 0.001) μm at 12 months, 146 ± 39 μm (*P* < 0.001) at 24 months, and 143 ± 61 μm (*P* < 0.001) at 36 months. The mean changes of CRT for 36 months were 225 ± 184.4 μm in typical AMD and 144 ± 118.1 μm in PCV (typical AMD: PCV, *P* = 0.162) (Fig. 3). The mean SFCT was 288 ± 97 μm at baseline, 243 ± 82 μm (*P* < 0.001) at 12 months, 228 ± 80 μm (*P* < 0.001) at 24 months, and 208 ± 63 μm (*P* < 0.01) at 36 months. The mean SFCT change for 36 months in PCV (− 82 ± 59 μm) was not significantly different from that in typical AMD (− 61 ± 44 μm, *P* = 0.579) (Fig. 4). The mean CRT and mean SFCT decreased significantly at 12, 24, and 36 months compared to those at baseline in both eyes with typical AMD and those with PCV.

Dry macula was achieved in 24 eyes (82.7%) at 12 months, 24 eyes (82.7%) at 24 months, and 23 eyes (79.3%) at 36 months. There were no significant differences between 12, 24, and 36 months in the presence of dry macula. Twenty eyes (69%) showing dry macula at 12 months (dry macula group) kept dry at 24 and 36 months. In addition, the mean number of injections was 16.1 ± 0.4 for 36 months in the dry macula group. Three eyes (10.3%) with exudation at 12 months remained with exudation at 24 and 36 months (Table 2). No patients switched to another drug or received photodynamic therapy during the follow-up period. The mean number of injections was 25.3 ± 4.0/36 months in the non-dry macular group. Three eyes (10.3%) showed atrophy of the retina at the end of the follow-up.

**Table 2:** Macular Findings

12M dry				12M wet			
24M dry		24M wet		24M dry		24M wet	
36M dry	36M wet	36M dry	36M wet	36M dry	36M wet	36M dry	36M wet
20	2	1	1	2	0	0	3
(69.0%)	(7.0%)	(3.5%)	(3.5%)	(6.9%)	(0%)	(0%)	(10.3%)

Wet: any exudative changes or hemorrhage except for retinal pigment epithelium detachment at the macula in fundus examination and optical coherence tomography. Dry: not wet

There were no ophthalmic or general adverse events, such as massive subretinal hemorrhage, cardiac infarction, or cerebellar infarction, during the three-year follow-up period. No patient discontinued treatment due to adverse events or poor treatment.

## Discussion

This study reported the prospective three-year results of aflibercept administration for nAMD. In the first year, all patients received a loading dose of IAI monthly for the first 3 months, and a fixed dose of IAI was administered every 2 months. Then, a TAE regimen based on four-week intervals was implemented in the following 2 years. In all patients, visual acuity improved significantly in the first year and was maintained in the third year. The exudative change was controlled for 36 months with fixed doses and a TAE regimen of aflibercept. Although anti-VEGF therapy improved visual acuity until the second year of treatment in this study, visual outcomes after the second year differed in previous reports [10–14]. The duration of the follow-up period, the treatment regimen, and the efficacy of drugs may be associated with the long-term visual outcome of anti-VEGF treatment [15]. Fixed dosing in the maintenance phase may be ideal for reducing the recurrence of exudative changes [16]. However, long-term fixed dosing may induce socioeconomic problems as well as patient and ophthalmologist burden. In contrast, as Kuroda et al. reported that dry macula was maintained for 1 year in 34% of the eyes after achieving dry macula by 3 months of loading doses when the eyes were treated with PRN [17], a personalized approach may resolve these problems.

TAE [18] treatment is administered to patients with nAMD as personalized treatment. The TAE approach can improve visual results and reduce the number of hospital visits compared to the PRN approach [19–21]. There have been a few reports on TAE regimens with aflibercept followed-up over 3 years.

Eleftheriadou et al. reported that aflibercept increased the mean visual acuity within 1 year with a similar regimen to that used for the first year in this study [18]. The visual gain was sustained for 3 years with a TAE regimen after the second year. The mean gain at 3 years was 6.6 letters in ETDRS, with statistical significance [14]. Traine et al. reported that aflibercept increased mean visual acuity with TAE without first-year fixed dosing (mean gain, + 5.7 letters in ETDRS). Although the mean visual acuity showed a decreasing tendency in the third year, the gain of visual acuity maintained statistical significance for 3 years (mean gain, + 4.4 letters in ETDRS) [22]. The visual gain (logMAR − 0.152) in our prospective study based on a four-week interval TAE regimen was similar to the results of the two retrospective studies based on a two-week interval TAE regimen [14, 22]. These results suggest that a reduction in the number of hospital visits for IAI from two-week to four-week intervals may not affect the visual gain in nAMD patients. The mean number of injections in the report by Eleftheriadou et al. was 15.9 times over 3 years, which was slightly less than that in the current study (17.2/three years) [14]. The prospective nature of the present study may be associated with the injection number, as the minimum number of injections in our study was 16 times over 3 years.

Retinal morphology typically improves rapidly after anti-VEGF therapy. A previously reported systemic review suggested greater retinal thickness changes in a TAE regimen than a PRN regimen [23]. Retinal thickness was reported to have rebounded after 1 year in a three-year ranibizumab monotherapy with a PRN regimen [8]. At the same time, it was sustained over 3 years in aflibercept monotherapy with a TAE regimen, both in this study and another previous report [22]. These findings indicate that a TAE regimen can provide long-term morphological stability compared to a PRN regimen.

Choroidal thickness reportedly decreases over 2 years in eyes treated with aflibercept [24–26]. The SFCT decreased after the second year in the current study, with the mean differences in SFCT being 45 μm (baseline-12 months), 15 μm (12 months–24 months), and 20 μm (24 months–36 months). In addition, the mean SFCT decrease with age was reported to be 2.98 μm/year in a linear regression model of healthy Japanese individuals [27]. The rate of SFCT decrease in nAMD eyes treated with aflibercept using a TAE regimen may exceed that in healthy individuals. A previous report suggested a relationship between SFCT decrease and macular atrophy [24]. Atrophy of the retina, retinal pigment epithelium, and choroid may be associated with the decline of visual functions in eyes with nAMD treated over the long term.

In this study, 82% of eyes achieved dry macula after 1 year of IAI with fixed intervals. Moreover, 83% of those eyes maintained dry macula throughout the three-year study period. Since TAE with aflibercept provided a high rate of morphological stability in nAMD, we should consider reducing the number of IAI in cases where long-term maintenance of over 2 years is required. In contrast, 10% of the eyes with exudation at 1 year remained exudated until the end of the study. These eyes were considered non-responders to aflibercept [28]. Alternative treatments, such as switching to other drugs or photodynamic therapy, should be considered in the early stage of treatment for these eyes. As most of the patients achieved dry macula and 3 months interval IAI at the end of the follow-up, we plan to introduce the “treat-extend-stop” protocol at four-year [29].

Limitations. First, the sample size of this study was small. Twenty-one patients did not completely follow up for 36 months. Although there were no specific reasons for incomplete follow-up, it may have influenced the results. However, the long-term prospective nature of this study can be worth discussing. Second, we did not monitor the nature of CNV using angiography. Therefore, we could not evaluate the long-term efficacy of aflibercept for neovascular membranes. We should use it in future studies since OCT angiography potentially evaluates the nature of CNV.

## Conclusion

Long-term aflibercept injection can achieve visual improvement and reduce the thickness of the retina and choroid in nAMD patients. Morphological improvement of these tissues may not be sufficient to sustain earlier visual improvement over the long-term.

## Data Availability

Data are available from Kanako Itagaki (n-kana@fmu.ac.jp) for researchers who meet the criteria for access to confidential data.

## Abbreviations

AMD: Age-related macular degeneration
CNV: Choroidal neovascularization
CRT: Central retinal thickness
IAI: Intravitreal aflibercept injection
nAM: Neovascular age-related macular degeneration
OCT: Optical coherence tomography
PCV: Polypoidal choroidal vasculopathy
PlGF: Placental growth factor
PRN: Pro re nata
SD: Standard deviation
SFCT: Subfoveal choroidal thickness
TAE: Treat-and-extend
VEGF: Vascular endothelial growth factor
VEGFR: Vascular endothelial growth factor receptor

## References

[1] Congdon N, O'Colmain B, Klaver CC, Klein R, Munoz B, Friedman DS, Kempen J, Taylor HR, Mitchell P (2004). Causes and prevalence of visual impairment among adults in the United States. Arch Ophthalmol.

[2] Macular Photocoagulation Study Group (1991). Laser photocoagulation of subfoveal neovascular lesions in age-related macular degeneration. Results of a randomized clinical trial. Arch Ophthalmol.

[3] Treatment of age-related macular degeneration with photodynamic therapy (TAP) Study Group (1999). Photodynamic therapy of subfoveal choroidal neovascularization in age-related macular degeneration with verteporfin: one-year results of 2 randomized clinical trials--TAP report. Arch Ophthalmol.

[4] Mauget-Faysse M, Chiquet C, Milea D, Romestaing P, Gerard JP, Martin P, Koenig F (1999). Long term results of radiotherapy for subfoveal choroidal neovascularisation in age related macular degeneration. Br J Ophthalmol.

[5] de Massougnes S, Dirani A, Mantel I (2018). Good visoual outcom at 1 year in neovascular age-related macular degeneration with pigment epithelium detachment: factors influencing the treatment response. Retina.

[6] Lalwani GA, Rosenfeld PJ, Fung AE, Dubovy SR, Michels S, Feuer W, Davis JL, Flynn HW, Esquiabro M (2009). A variable-dosing regimen with intravitreal ranibizumab for neovascular age-related macular degeneration: year 2 of the PrONTO Study. Am J Ophthalmol.

[7] Brynskov T, Munch IC, Larsen TM, Erngaard L, Sorensen TL (2020). Real-world 10-year experiences with intravitreal treatment with ranibizumab and aflibercept for neovascular age-related macular degeneration. Acta Ophthalmol.

[8] Wada I, Oshima Y, Shiose S, Kano K, Nakao S, Kaizu Y, Yoshida S, Ishibashi T, Sonoda KH (2019). Five-year treatment outcomes following intravitreal ranibizumab injections for neovascular age-related macular degeneration in Japanese patients. Graefes Arch Clin Exp Ophthalmol.

[9] Veritti D, Sarao V, Missiroli F, Ricci F, Lanzetta P (2019). Twelve-month outcomes of intravitreal aflibercept for neovascular age-related macular degeneration: fixed versus as-needed dosing. Retina..

[10] Rofagha S, Bhisitkul RB, Boyer DS, Sadda SR, Zhang K (2013). Seven-year outcomes in ranibizumab-treated patients in ANCHOR, MARINA, and HORIZON: a multicenter cohort study (SEVEN-UP). Ophthalmology.

[11] Bhisitkul RB, Mendes TS, Rofagha S, Enanoria W, Boyer DS, Sadda SR, Zhang K (2015). Macular atrophy progression and 7-year vision outcomes in subjects from the ANCHOR, MARINA, and HORIZON studies: the SEVEN-UP study. Am J Ophthalmol.

[12] Berg K, Roald AB, Navaratnam J, Bragadottir R (2017). An 8-year follow-up of anti-vascular endothelial growth factor treatment with a treat-and-extend modality for neovascular age-related macular degeneration. Acta Ophthalmol.

[13] Kaiser PK, Singer M, Tolentino M, Vitti R, Erickson K, Saroj N, Berliner AJ, Chu KW, Zhu X, Williams Liu Z (2017). Long-term safety and visual outcome of intravitreal aflibercept in neovascular age-related macular degeneration: VIEW 1 extension study. Ophthalmology retina.

[14] Eleftheriadou M, Gemenetzi M, Lukic M, Sivaprasad S, Hykin PG, Hamilton RD, Rajendram R, Tufail A, Patel PJ (2018). Three-year outcomes of aflibercept treatment for neovascular age-related macular degeneration: evidence from a clinical setting. Ophthalmol Ther.

[15] Bakri SJ, Thorne JE, Ho AC, Ehlers JP, Schoenberger SD, Yeh S, Kim SJ (2019). Safety and efficacy of anti-vascular endothelial growth factor therapies for neovascular age-related macular degeneration: a report by the American Academy of ophthalmology. Ophthalmology.

[16] Peden MC, Suner IJ, Hammer ME, Grizzard WS (2015). Long-term outcomes in eyes receiving fixed-interval dosing of anti-vascular endothelial growth factor agents for wet age-related macular degeneration. Ophthalmology.

[17] Kuroda Y, Yamashiro K, Miyake M, Yoshikawa M, Nakanishi H, Oishi A, Tamura H, Ooto S, Tsujikawa A, Yoshimura N (2015). Factors associated with recurrence of age-related macular degeneration after anti-vascular endothelial growth factor treatment : a retrospective cohort study. Ophthalmology.

[18] Engelbert M, Zweifel SA, Freund KB (2010). Long-term follow-up for type 1 (subretinal pigment epithelium) neovascularization using a modified “treat and extend” dosing regimen of intravitreal antivascular endothelial growth factor therapy. Retina.

[19] Augsburger M, Sarra GM, Imesch P (2019). Treat and extend versus pro re nata regimens of ranibizumab and aflibercept in neovascular age-related macular degeneration: a comparative study. Graefes Arch Clin Exp Ophthalmol.

[20] Arnold JJ, Campain A, Barthelmes D, Simpson JM, Guymer RH, Hunyor AP, McAllister IL, Essex RW, Morlet N, Gillies MC (2015). Two-year outcomes of “treat and extend” intravitreal therapy for neovascular age-related macular degeneration. Ophthalmology.

[21] Ghosh W, Wickstead R, Claxton L, Kusel J, Taylor M, Fleetwood K, Pulikottil-Jacob R (2016). The cost-effectiveness of ranibizumab treat and extend regimen versus aflibercept in the UK. Adv Ther.

[22] Traine PG, Pfister IB, Zandi S, Spindler J, Garweg JG (2019). Long-term outcome of intravitreal aflibercept treatment for neovascular age-related macular degeneration using a “treat-and-extend” regimen. Ophthalmol Retina.

[23] Chin-Yee D, Eck T, Fowler S, Hardi A, Apte RS (2016). A systematic review of as needed versus treat and extend ranibizumab or bevacizumab treatment regimens for neovascular age-related macular degeneration. Br J Ophthalmol.

[24] Matsumoto H, Morimoto M, Mimura K, Ito A, Akiyama H (2018). Treat-and-extend regimen with Aflibercept for Neovascular age-related macular degeneration. Ophthalmol Retina.

[25] Barakat A, Rufin V, Tran THC. Two year outcome in treatment-naive patients with neovascular age-related macular degeneration (nAMD) using an individualized regimen of Aflibercept. J Ophthalmol. 2018;41(7):603–10. 10.1016/j.jfo.2018.01.005 30166233

[26] Koizumi H, Kano M, Yamamoto A, Saito M, Maruko I, Sekiryu T, Okada AA, Iida T (2015). Subfoveal choroidal thickness during aflibercept therapy for neovascular age-related macular degeneration: twelve-month results. Ophthalmology.

[27] Wakatsuki Y, Shinojima A, Kawamura A, Yuzawa M (2015). Correlation of aging and segmental choroidal thickness measurement using swept source optical coherence tomography in healthy eyes. PLoS One.

[28] Hara C, Wakabayashi T, Toyama H, Fukushima Y, Sayanagi K, Sato S, Sakaguchi H, Nishida K. Characteristics of patients with neovascular age-related macular degeneration who are non-responders to intravitreal aflibercept. Br J Ophthalmol. 2018;bjophthalmol-2018-312275. 10.1136/bjophthalmol-2018-312275. 10.1136/bjophthalmol-2018-312275 29907628

[29] Adrean SD, Chaili S, Grant S (2018). Pirouz a:recurrence rate of choroidal neovascularization in neovascular age-related macular degeneration managed with a treat-extend-stop protocol. Ophthalmol Retina.

